# Addition of Induction or Consolidation Chemotherapy in Definitive Concurrent Chemoradiotherapy Versus Concurrent Chemoradiotherapy Alone for Patients With Unresectable Esophageal Cancer: A Systematic Review and Meta-Analysis

**DOI:** 10.3389/fonc.2021.665231

**Published:** 2021-09-13

**Authors:** Jianing Wang, Linlin Xiao, Shuai Wang, Qingsong Pang, Jun Wang

**Affiliations:** ^1^Department of Radiation Oncology, The Fourth Hospital of Hebei Medical University, Hebei Clinical Research Center for Radiation Oncology, Shijiazhuang, China; ^2^Department of Radiation Oncology, National Clinical Research Center for Cancer, Tianjin’s Clinical Research Center for Cancer, Key Laboratory of Cancer Prevention and Therapy, Tianjin Medical University Cancer Institute and Hospital, Tianjin, China

**Keywords:** concurrent chemoradiotherapy, induction chemotherapy, consolidation chemotherapy, meta-analysis, esophageal cancer

## Abstract

**Background:**

Concurrent chemoradiotherapy (CCRT) has become the standard of care in esophageal carcinoma patients who are not surgical candidates. The efficacy of induction chemotherapy (IC) or consolidation chemotherapy (CCT) for unresectable esophageal cancer (EC) treated with CCRT is unclear. We performed a systematic review and meta-analysis of published papers to evaluate the potential benefit of IC or CCT for patients with EC.

**Methods:**

Eligible studies of IC followed by CCRT (IC-CCRT) *vs*. CCRT alone or CCRT followed by CCT (CCRT-CCT) *vs*. CCRT alone were retrieved through extensive searches of the PubMed, Science Direct, Embase, and Cochrane Library databases from the establishment of the database to July 31, 2021. Data such as 1-, 2-, 3-, and 5-year overall survival (OS), local recurrence rate (LRR), and distant metastasis rate (DMR) were collected for meta-analysis to evaluate the efficacy of IC/CCT.

**Results:**

Four studies of IC-CCRT *vs*. CCRT including 836 EC patients and six studies of CCRT-CCT *vs*. CCRT including 1,339 patients with esophageal squamous cell carcinoma (ESCC) were finally identified in our analysis. Both IC-CCRT group [hazard ratio (HR) 0.446, 95% CI 0.286–0.693; p < 0.001] and CCRT-CCT group (HR 0.542, 95% CI 0.410–0.716; p < 0.001) exhibited statistically significant improvement in 1-year OS rate compared to that of CCRT, while the 2-year OS rate of IC-CCRT (HR 0.803, 95% CI 0.589–1.095; p = 0.166) or CCRT-CCT (HR 0.783, 95% CI 0.600–1.022; p = 0.072) was similar with that of CCRT. And the 3-year OS rate between IC-CCRT and CCRT was similar (HR 1.065, 95% CI 0.789–1.439; p = 0.680). However, comparing with CCRT alone, the CCRT-CCT group had lower DMR [odds ratio (OR) 1.562, 95% CI 1.090–2.240; p = 0.015] and higher 3-year OS rate (HR 0.786, 95% CI 0.625–0.987; p = 0.039). Besides, no differences were observed between the CCRT-CCT and CCRT groups in 5-year OS rate (HR 0.923, 95% CI 0.706–1.205; p = 0.555) and LRR (OR 0.899, 95% CI 0.686–1.179; p = 0.441).

**Conclusion:**

The study revealed the short-time survival benefit of additional IC or CCT compared to CCRT alone for patients with unresectable EC, and CCRT followed by CCT could significantly reduce the risk of distant metastases.

## Introduction

Radiotherapy plays an important role in the comprehensive treatment of esophageal carcinoma. The RTOG 85-01 trial has demonstrated that concurrent chemoradiotherapy (CCRT) is superior to radiation alone, with a favorable long-term survival for nonoperative esophageal cancer (EC) patients (1). Although CCRT is the standard treatment for patients with locally advanced disease who are not surgical candidates or refuse surgery, the local recurrence rate (LRR) of CCRT remains up to 40%–60%, and the 5-year survival rate is extremely low once recurrence occurs (2, 3). Therefore, several intensified treatment modalities have been attempted to improve treatment outcomes, such as the addition of induction chemotherapy (IC) or consolidation chemotherapy (CCT).

Theoretically, the additional IC followed by CCRT (IC-CCRT) has potential benefit on response rate, early eradication of micrometastases, increased tumor radiosensitivity, and even prolonged overall survival (OS) because of tumor shrinkage (4). Byfield et al. (5) conducted the first clinical trial of IC followed by radiotherapy alone for EC patients in the 1980s, which achieved inspiring results. In contrast, Chen et al. (6) concluded that the additional IC failed to prolong OS, locoregional failure-free survival, or distant failure-free survival compared with CCRT alone.

CCT is defined as prolonged chemotherapy duration by administration of additional drugs at the end of a defined number of initial chemotherapy cycles after achieving a maximum tumor response in an individual patient. The rationale for CCT is based on a hypothesis stating that the early use of non-cross-resistant agents might increase the probability of killing more cancer cells (7). To date, several studies have been conducted to explore the efficacy of CCT for non-small-cell lung cancer patients, which all demonstrated that these management strategies were ineffective (8–10). Actually, patients received CCT following CCRT in RTOG 85-01 trials (1); the reason why RTOG investigators added CCT to CRT had not been clarified. Up to now, the reports on CCRT followed by CCT (CCRT-CCT) were rare for EC patients, and the results were conflicting.

Due to a few publications, inconsistent conclusions, and lack of prospective randomized controlled studies, the value of the addition of IC or CCT for EC patients remains controversial. Thus, our study aimed to evaluate the outcomes of IC-CCRT or CCRT-CCT *vs*. CCRT alone in unresectable EC by means of a systematic review and meta-analysis, which could further provide better clinical guidance for the treatment of EC.

## Methods

### Search Strategy

The PubMed, Science Direct, Embase, and Cochrane Library databases were searched from their inception to July 31, 2021. Search keywords included “esophageal or oesophageal” and “carcinoma or cancer or neoplasm” and “induction chemotherapy or neoadjuvant chemotherapy” and “consolidation or adjuvant chemoradiotherapy” and “concurrent or definitive chemoradiotherapy.” Additional papers were retrieved through hand-searching. The search was limited to human subjects and English-language published articles.

### Inclusion Criteria and Exclusion Criteria

The inclusion criteria included the following: (1) studies that involved patients who had unresectable EC; (2) studies comparing the effects of IC-CCRT or CCRT-CCT *vs*. CCRT alone; (3) the hazard ratio (HR), odds ratio (OR), and 95% confidence intervals (CIs) in these studies were reported or could be calculated; (4) randomized controlled trials (RCTs) and cohort studies.

The exclusion criteria included the following: (1) studies that did not include survival data as endpoints; (2) studies whose patients underwent sequential treatments, neoadjuvant or adjuvant CRT combined with surgery, palliative CRT or radiotherapy alone; (3) studies only published as a letter or conference paper; (4) repeated reports on survival data of the same population; (5) studies that involved specific elderly population.

### Quality Evaluation

Case-control study evaluation guidelines were applied in order to evaluate the quality of each manuscript for the following criteria: 1) whether gender, age, and tumor location were clearly stated; 2) whether the comparability of the two groups was analyzed; 3) whether the statistical method was appropriate; 4) whether the test was designed as a prospective randomized control study; 5) whether biases were discussed in the study. A score was assigned for each of the five items, with a total score of ≥3 indicative of reliable quality. Two researchers independently reviewed the literature according to the unified quality standard, with results cross-checked. If there were some different opinions, a third researcher would be invited to solve the disagreement. The systematic review was conducted in accordance with the Preferred Reporting Items for Systematic Reviews and Meta-Analyses (PRISMA) guidelines (11).

### Outcome Measures

The outcome measures of this study were 1-, 2-, 3-, and 5-year OS rate, LRR, and distant metastasis rate (DMR) to examine the efficiency of various management strategies.

### Statistical Analysis

Data were analyzed using Stata version 12.0. HR, OR, and 95% CI were used to measure the effect. Heterogeneity was assessed using the Q test. Statistical significance was set at p < 0.05. If there was a significant heterogeneity (p ≤ 0.05), the random-effects model was adopted; otherwise, a fixed-effects model was used (p > 0.05). The combined effect size was tested by the z test. Funnel plots were created to evaluate the risk of publication bias.

## Results

### Literature Search and Study Selection

**Figure 1:**
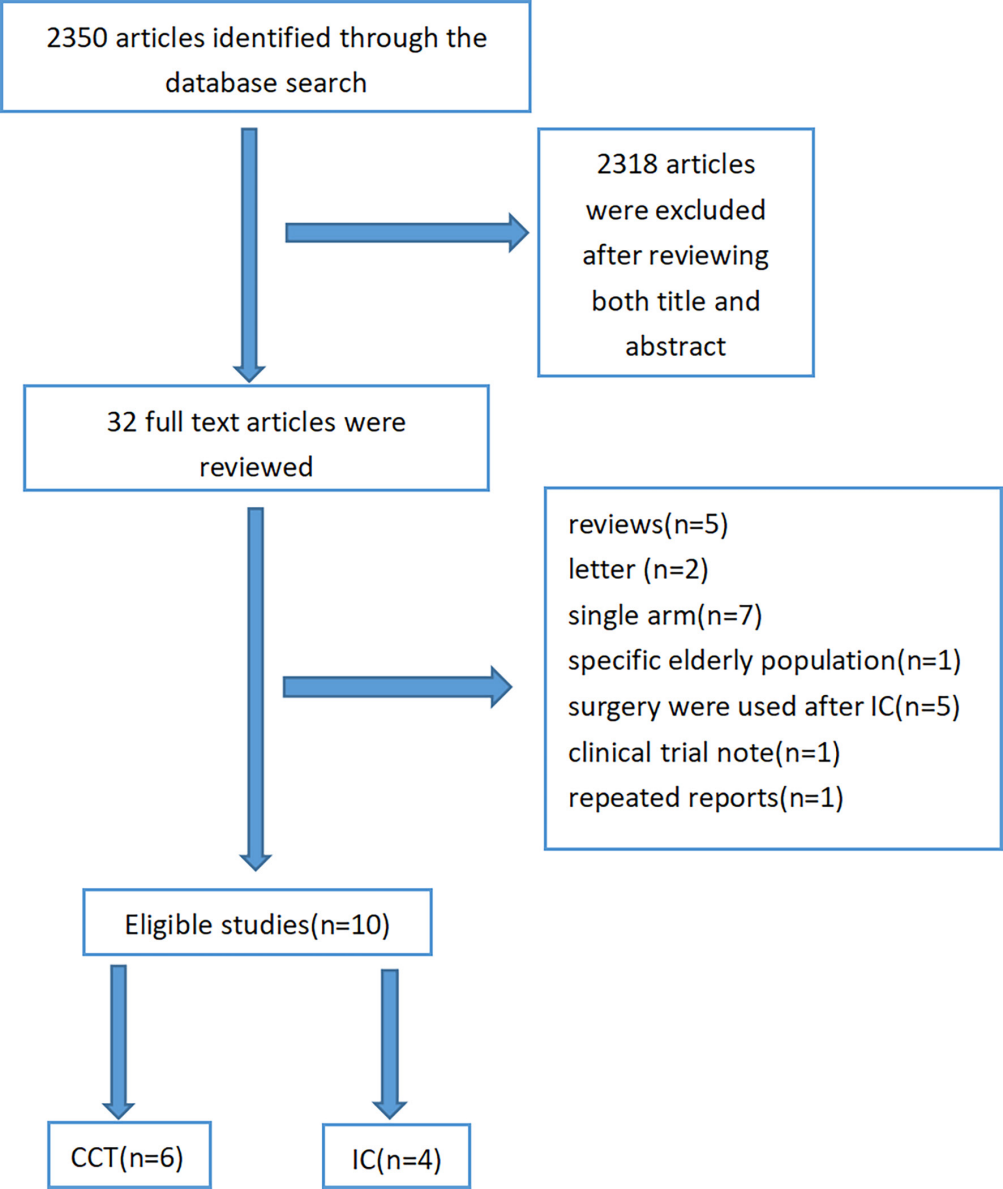
Study selection process.

After excluding 2,340 articles from a total of 2,350 citations, finally, 10 articles were subjected to our meta-analysis. A detailed study flow diagram is shown in **Figure 1**. Among the 10 articles, there were four studies including three retrospective studies and one prospective study that compared IC-CCRT with CCRT and six other retrospective studies that compared CCRT-CCT with CCRT (6, 12–20) (**Tables 1**, **2**). The number of patients within these four studies on IC-CCRT *vs*. CCRT was 836, including 486 esophageal squamous cell carcinoma (ESCC) patients and 350 esophageal adenocarcinoma (EAC) patients. A total of 1,339 patients were included in the six other studies on CCRT-CCT *vs*. CCRT whose pathological types were all ESCC.

**Table 1:** Basic characteristics of the included studies (IC-CCRT *vs*. CCRT alone group).

Author	Year	Stage	ESCC /EAC	IC-CCRT/ CCRT alone	Radiation dose (Gy)	Current chemotherapy regimen	Induction chemotherapy	The cycles of IC	Quality evaluation
Chen et al. (6)	2017	II–IV	60/0	41/19	50.4–66	PF/TP/P/C/F	PF/TP/C	1–3	4
Luo et al. (12)	2017	III–IV	170/0	85/85	60 (50–70)	DP	DP	2	4
Xi et al. (13)	2017	IB–III	146/350	162/334	50.4 (41.4–66)	PTF/PF/TP/TF	PTF/PF/TP/TF	2–4	4
Liu et al. (14)	2021	IIA–IVA	110/0	55/55	60	DP	DP	2	5

ESCC, esophageal squamous cell carcinoma; EAC, esophageal adenocarcinoma; DP, docetaxel plus cisplatin; TF, platinum plus taxane; PF, platinum plus fluorouracil; C, capecitabine; PTF, fluoropyrimidine, platinum plus taxane; PF, platinum plus fluoropyrimidine; TP, platinum plus taxane; TF, fluoropyrimidine plus taxane; IC, induction chemotherapy; CCRT, concurrent chemoradiotherapy.

**Table 2:** Basic characteristics of the included studies (CCRT-CCT *vs*. CCRT alone group).

Author	Year	Stage	ESCC/EAC	CCRT-CCT/CCRT alone	Radiation dose (Gy)	Current chemotherapy regimen	CCT regimen	The cycles of CCT	Quality evaluation
Wu et al. (15)	2017	I–III	209/0	67/142	≥50.4	PF	PF/ND/DP	2	4
Chen et al. (16)	2018	II–III	524/0	262/262	50.4–66	PF	PF	2	4
Chen et al. (17)	2018	II–IVB	187/0	89/98	50–66	PF/TP	PF/TP	2 (1–4)	4
Chen et al. (18)	2018	I–IV	124/0	65/59	50–74	PF/PP	PF/P	2–4	4
Koh et al. (19)	2020	I–III	73/0	56/17	50–70	PF/P/F	PF/P/F	–	4
Zhang et al. (20)	2020	I–III	222/0	113/109	50.4–66	LFP/PF/TP	LFP/PF/TP	1–4	4

ESCC, esophageal squamous cell carcinoma; EAC, esophageal adenocarcinoma; P, cisplatin; F, fluorouracil; PF, cisplatin plus fluorouracil; DP, docetaxel plus cisplatin; ND, nedaplatin plus cisplatin; PP, paclitaxel plus cisplatin; LFP, cisplatin plus 5-fluorouracil plus calcium folinate; TP, cisplatin plus paclitaxel; m, months; CCT, consolidation chemotherapy; CCRT, concurrent chemoradiotherapy.

### Outcome Data

#### Induction Chemotherapy Followed by Concurrent Chemoradiotherapy *vs*. Concurrent Chemoradiotherapy

**Figure 2:**
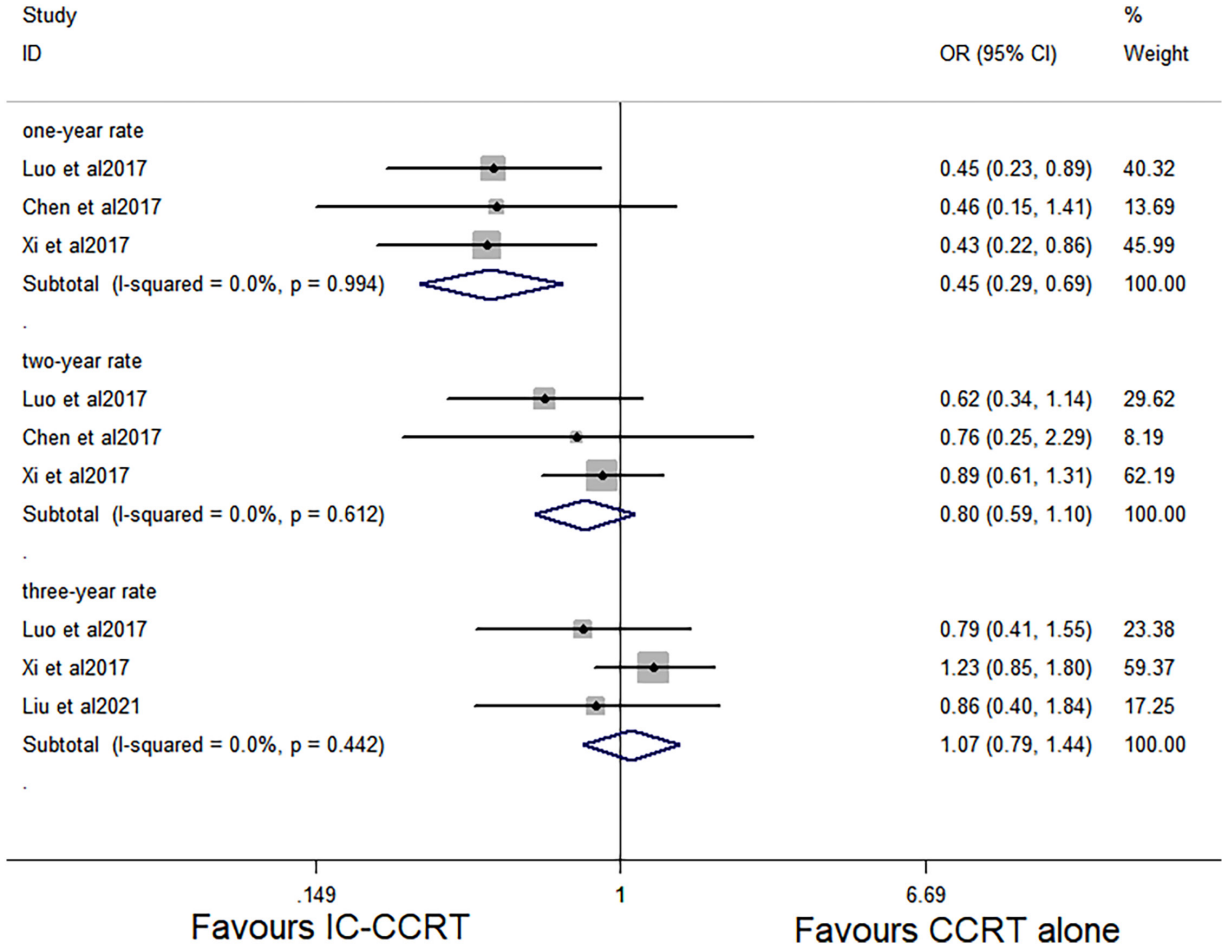
Effects of IC-CCRT *vs*. CCRT alone on 1-, 2-, and 3-year OS. IC-CCRT, induction chemotherapy followed by concurrent chemoradiotherapy; CCRT, concurrent chemoradiotherapy; OS, overall survival; CI, confidence interval; ES, effect size.

**Figure 3:**
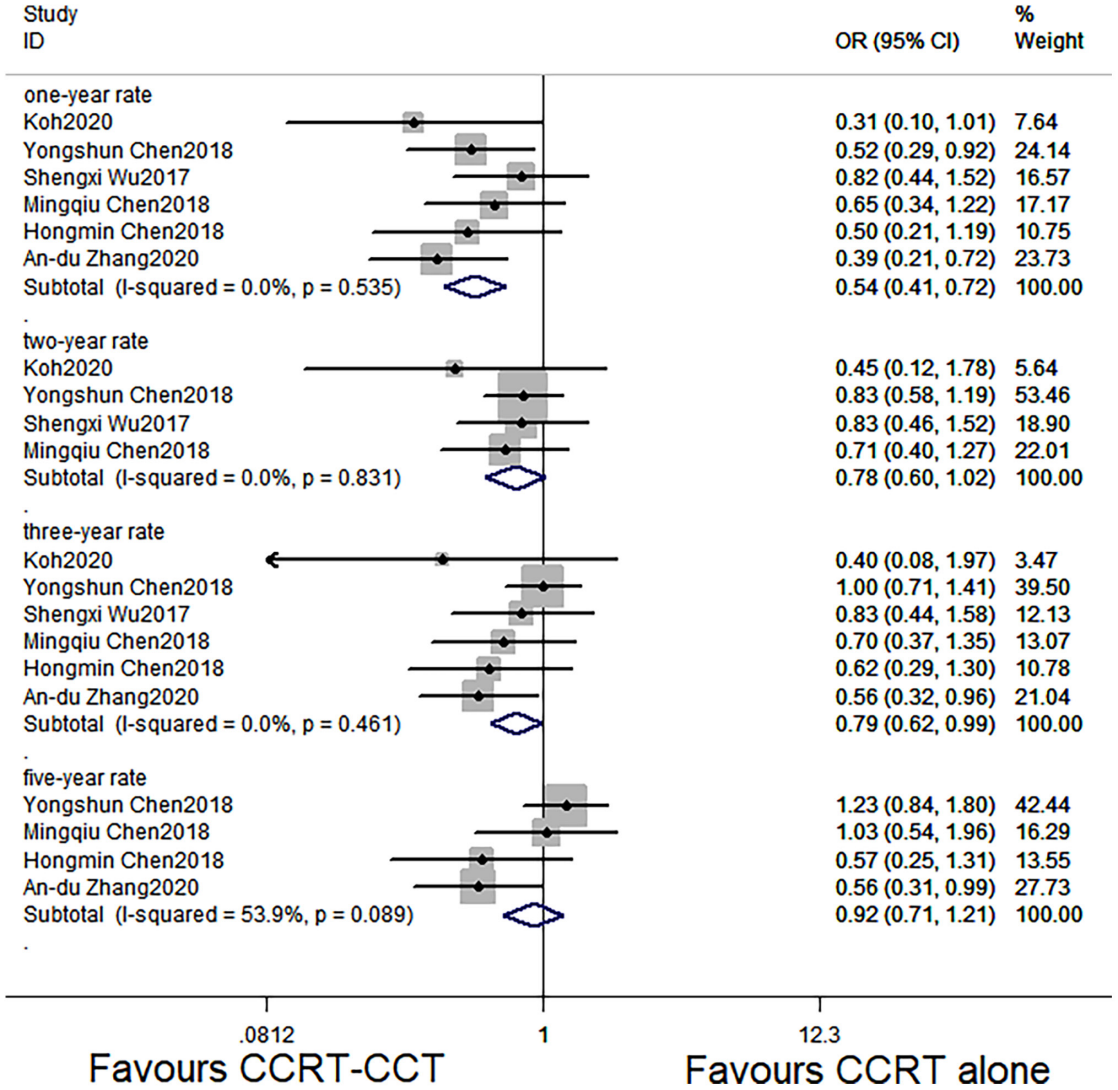
Effects of CCRT-CCT *vs*. CCRT alone on 1-, 2-, 3-, and 5-year OS. CCRT-CCT, concurrent chemoradiotherapy followed by consolidation chemotherapy; CCRT, concurrent chemoradiotherapy; OS, overall survival; CI, confidence interval; ES, effect size.

Data regarding 1-year and 2-year OS rate were available in the three studies with 726 patients (6, 12, 13). The 1-year OS rate was significantly higher in patients who were treated with IC-CCRT (HR 0.445, 95% CI 0.286–0.693; p < 0.001; **Figure 2**). In addition, three studies analyzed 3-year OS rate of the two groups (12–14). There was no significant difference in 2-year OS rate (HR 0.803, 95% CI 0.589–1.095; p = 0.166; **Figure 2**) and 3-year OS rate (HR 1.065, 95% CI 0.789–1.439; p = 0.680; **Figure 2**) between the two groups.

#### Concurrent Chemoradiotherapy Followed by Consolidation Chemotherapy *vs*. Concurrent Chemoradiotherapy

The six studies with 1,339 patients all reported 1- and 3-year OS rates of the CCRT-CCT group and CCRT group (15–20). Four articles reported 2-year OS rate of the two groups (15–17, 19). Four studies (16–18, 20) analyzed 5-year OS rate, and three studies (15–17) analyzed LRR and DMR of the two groups.

**Figure 4:**
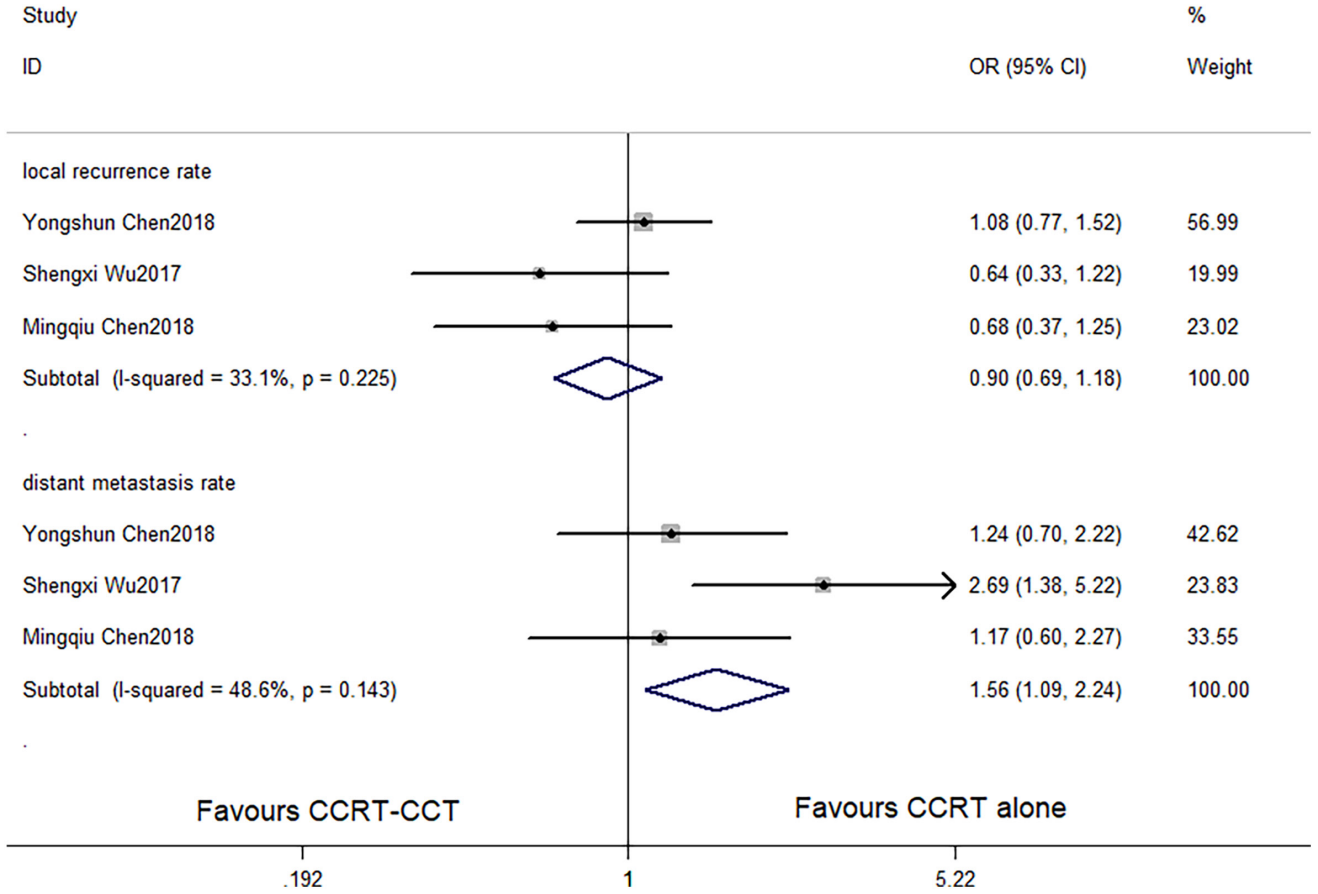
Effects of CCRT-CCT *vs*. CCRT alone on local recurrence rate and distant metastasis rate. CCRT-CCT, concurrent chemoradiotherapy followed by consolidation chemotherapy; CCRT, concurrent chemoradiotherapy; CI, confidence interval; OR, odds ratio.

CCRT-CCT group had a significant advantage over the CCRT group in 1-year OS rate (HR 0.542, 95% CI 0.410–0.716; p < 0.001; **Figure 3**) and 3-year OS rate (HR 0.786, 95% CI 0.625–0.987; p = 0.039; **Figure 3**). The CCRT-CCT group had lower DMR (OR 1.562, 95% CI 1.090–2.240; p = 0.015; **Figure 4**). However, no differences were seen between the two groups in 2-year OS rate (HR 0.783, 95% CI 0.600–1.022; p = 0.072; **Figure 3**), 5-year OS rate (HR 0.923, 95% CI 0.706–1.205; p = 0.555; **Figure 3**), and LRR (OR 0.899, 95% CI 0.686–1.179; p = 0.441; **Figure 4**).

### Sensitivity Analysis

Sensitivity analysis showed that the new combined HR/OR was similar with the original HR/OR (**Tables 3**, **4**).

**Table 3:** Sensitivity analysis for 1-, 2-, and 3-year OS rate (IC-CCRT *vs*. CCRT group).

Items	Study omitted	Estimate	95% CI	
One-year OS rate	Chen et al. (6)	0.525	0.357	0.774
	Luo et al. (12)	0.513	0.318	0.830
	Xi et al. (13)	0.587	0.397	0.869
	Combined	0.540	0.384	0.760
Two-year OS rate	Chen et al. (6)	0.886	0.737	1.064
	Luo et al. (12)	0.929	0.754	1.143
	Xi et al. (13)	0.811	0.625	1.053
	Combined	0.886	0.745	1.052
Three-year OS rate	Luo et al. (12)	1.067	0.914	1.245
	Xi et al. (13)	0.938	0.797	1.104
	Liu et al. (14)	1.043	0.913	1.192
	Combined	1.026	0.908	1.160

IC, induction chemotherapy; CCRT, concurrent chemoradiotherapy; OS, overall survival.

**Table 4:** Sensitivity analysis for 1-, 2-, 3-, and 5-year OS rate, LRR, and DMR (CCRT *vs*. CCRT-CCT group).

Items	Study omitted	Estimate	95% CI	
One-year OS rate	Wu et al. (15)	0.588	0.467	0.741
	Chen et al. (16)	0.667	0.538	0.826
	Chen et al. (17)	0.625	0.501	0.780
	Chen et al. (18)	0.651	0.528	0.802
	Koh et al. (19)	0.649	0.522	0.805
	Zhang et al. (20)	0.687	0.551	0.857
	Combined	0.644	0.528	0.786
Two-year OS rate	Wu et al. (15)	0.872	0.745	1.020
	Chen et al. (16)	0.888	0.765	1.030
	Chen et al. (17)	0.896	0.770	1.042
	Koh et al. (19)	0.894	0.777	1.028
	Combined	0.888	0.779	1.011
Three-year OS rate	Wu et al. (15)	0.907	0.825	0.998
	Chen et al. (16)	0.873	0.793	0.961
	Chen et al. (17)	0.915	0.831	1.007
	Chen et al. (18)	0.923	0.843	1.009
	Koh et al. (19)	0.918	0.840	1.003
	Zhang et al. (20)	0.941	0.858	1.032
	Combined	0.914	0.840	0.995
Five-year OS rate	Chen et al. (16)	0.902	0.810	1.004
	Chen et al. (17)	0.971	0.893	1.056
	Chen et al. (18)	0.994	0.912	1.078
	Zhang et al. (20)	1.017	0.935	1.106
	Combined	0.977	0.906	1.054
LRR	Wu et al. (15)	0.965	0.717	1.299
	Chen et al. (16)	0.660	0.424	1.028
	Chen et al. (17)	0.965	0.712	1.306
	Combined	0.899	0.686	1.179
DMR	Wu et al. (15)	1.211	0.782	1.874
	Chen et al. (16)	1.799	1.134	2.855
	Chen et al. (17)	1.761	1.145	2.709
	Combined	1.562	1.090	2.240

CCT, consolidation chemotherapy; CCRT, concurrent chemoradiotherapy; DMR, distant metastasis rate; LRR, local recurrence rate; OS, overall survival.

### Publication Bias Analysis

**Figure 5:**
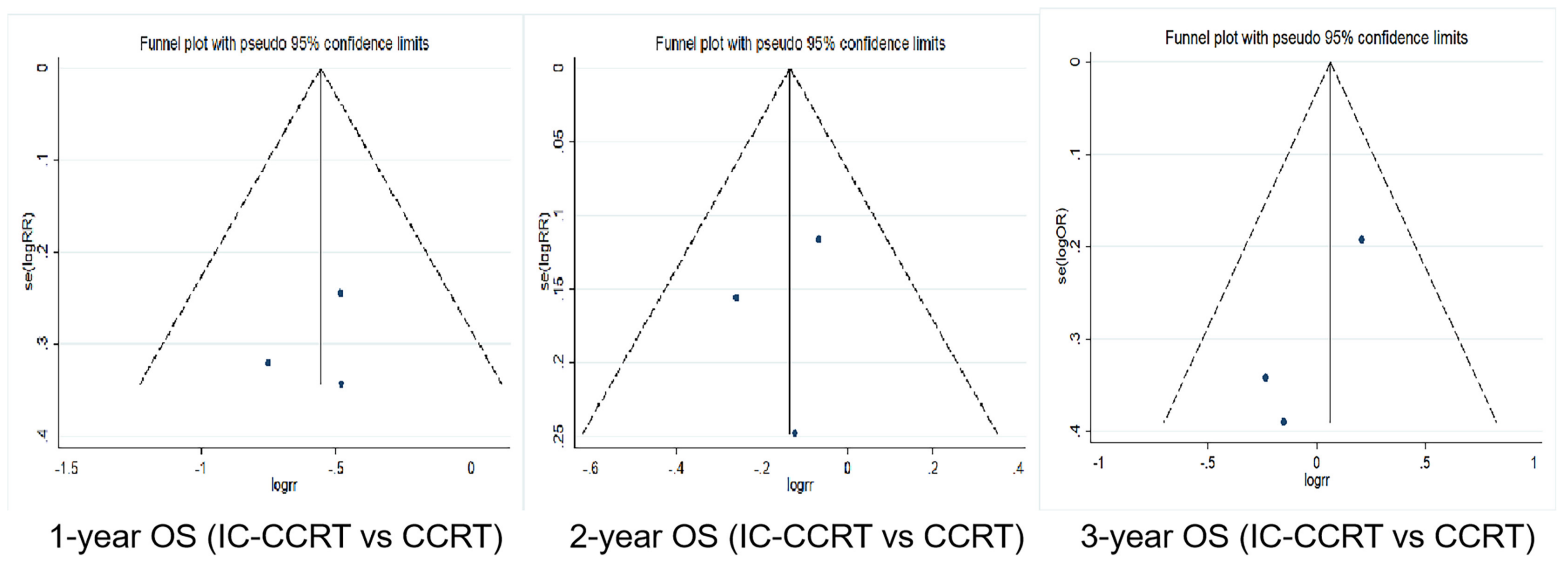
Funnel plots for 1-, 2-, and 3-year OS rate between IC-CCRT group and CCRT alone group. OS, overall survival; IC-CCRT, induction chemotherapy followed by concurrent chemoradiotherapy; CCRT, concurrent chemoradiotherapy; CCRT, concurrent chemoradiotherapy.

**Figure 6:**
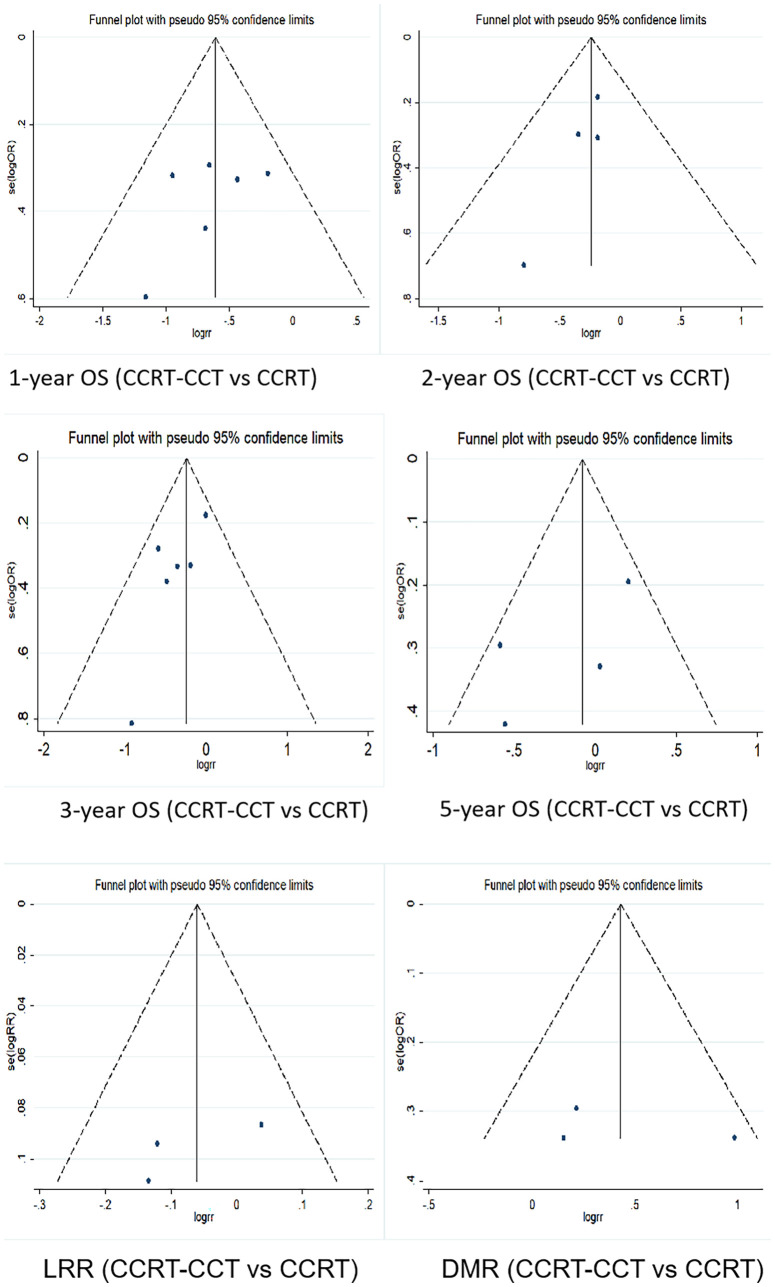
Funnel plots for 1-, 2-, 3-, and 5-year OS rate, LRR, and DMR between CCRT-CCT group and CCRT alone group. OS, overall survival; LRR, local recurrence rate; DMR, distant metastasis rate; CCRT-CCT, concurrent chemoradiotherapy followed by consolidation chemotherapy; CCRT, concurrent chemoradiotherapy.

Funnel plot was used to evaluate the publication bias. Egger’s regression test was conducted to analyze the symmetry of the funnel plot. None of the articles demonstrated publication bias (p > 0.05) (**Figures 5**, **6** and **Tables 5**, **6**).

**Table 5:** Publication bias results of selected articles (IC-CCRT *vs*. CCRT group).

Items	t	95% CI		p
One-year OS rate	-0.47	-32.863	30.511	0.719
Two-year OS rate	-0.40	-24.901	23.382	0.758
Three-year OS rate	-3.29	-11.006	6.476	0.188

IC, induction chemotherapy; CCRT, concurrent chemoradiotherapy; OS, overall survival.

**Table 6:** Publication bias results of selected articles (CCRT-CCT *vs*. CCRT group).

Items	t	95% CI		p
One-year OS rate	-1.00	-6.527	3.070	0.374
Two-year OS rate	-2.66	-2.785	0.657	0.117
Three-year OS rate	-2.36	-3.792	0.304	0.077
Five-year OS rate	-1.56	-12.670	5.927	0.259
Local recurrence rate	-1.42	-74.957	59.923	0.391
Distant metastasis rate	0.53	-190.069	206.648	0.689

CCT, consolidation chemotherapy; CCRT, concurrent chemoradiotherapy; OS, overall survival.

## Discussion

Our meta-analysis evaluated the value of additional IC or CCT in unresectable EC undergoing CCRT. The results showed that the additional IC or CCT may be an important factor affecting short-term survival but not long-term survival. To the best of our knowledge, this is the first meta-analysis to discuss the efficacy of IC in patients with unresectable EC treated with CCRT.

In theory, the addition of IC before CCRT has the potential advantage to degrade the stage and reduce the size of tumor. Chemotherapy response before radiotherapy is associated with reduced lung and heart irradiation and increased tolerance of the patients. Lu et al. (21) reported that additional IC for EC patients treated with neoadjuvant CCRT followed by esophagectomy was associated with a higher pathological complete response (pCR) rate (45.3% *vs*. 33.5%, p = 0.005) and better 5-year OS rate (90.5% *vs*. 48.1%, p = 0.015) compared to CCRT alone. Recently a multicenter randomized phase II trial showed that adding IC prior to trimodality therapy did not improve pCR but was associated with longer median OS (22). In our study, the IC-CCRT group had a higher 1-year OS rate over that of the CCRT group (HR 0.445, 95% CI 0.286–0.693; p < 0.001), but 2- and 3-year OS rate between the two groups was similar. Several possible reasons may explain this result. Firstly, our study focused on nonoperative EC patients; the treatment modality might influence the prognosis. Secondly, the additional IC might facilitate the elimination of occult micrometastasis for the patients with well response to chemoradiotherapy, whereas it might not carry out a positive impact for poorly responding patients and seemed to have limited benefits in long-term survival. In the study by Luo et al. (12), the IC responders (51%) achieved significantly more favorable OS compared with the IC nonresponder group and the CCRT alone group (p = 0.002), and there was no significant difference in terms of OS between the IC-CCRT and CCRT groups. Liu et al. (14) also observed that patients who responded to IC had improved survival in the *post-hoc* analysis. Thirdly, histological types and tumor biological characteristics between ESCC and EAC are heterogeneous. Xi et al. (13) accounted for 41.9% of the total number of patients in the meta-analysis, and its negative results might impact the final analysis outcomes. However, in the sensitivity analysis after deleting this article by Xi et al. (13), the new results were consistent with the original results. How to predict the sensitivity to chemotherapy might be the decisive factor. Greally et al. (23) evaluated the predictive value of PET following IC in ESCC patients and assessed the impact of changing chemotherapy during radiation in PET nonresponders. They found that PET after IC highly predicts outcomes in ESCC patients who receive chemoradiation. Early predictions of the efficacy of induction therapy are imminent and needed to further clinical research.

In this meta-analysis, the patients included in the studies of CCRT-CCT *vs*. CCRT were all with ESCC. It was noteworthy that there were fewer patients developing distant metastases in the CCRT-CCT group than in the CCRT group [15 of 67 (22.4%) *vs*. 62 of 142 (43.7%), p = 0.003] in the study of Wu et al. (15), and the CCRT-CCT group had remarkably prolonged median OS of 26.4 months (p = 0.04), while the DMR and OS showed no differences in two other studies. In order to identify patients who may benefit from CCT, Chen et al. (17) conducted further analysis based on various prognostic factors, including clinical N-stage (N0, N1, and N2), clinical M-stage (M0 and M1), and so on, and no significant survival differences between CCRT-CCT and CCRT were observed in any subgroup. In our meta-analysis, the addition of CCT reduced the DMR (p = 0.015) and can improve 1- and 3-year OS rate. Koh et al. (19) validated the positive effect of intensified treatment on survival in ESCC patients with definitive CCRT by modern radiotherapy techniques, and 45.2% patients received radiotherapy with biologically effective dose (BED) ≥70 Gy. As the radiation doses in the involved studies were not uniform, whether the additional CCT is the main factor affecting prognosis needs to be further explored. Also, whether patients who responded poorly to CCRT could gain a survival benefit from CCT needs to be studied further. Kim et al. (24) divided patients who received CCT after CCRT into “good risk” and “poor risk” groups based on smoking >20 pack-years, dysphagia scores >2, and so on. In the “good risk” group, patients with following CCT showed better OS than those without (30.4 *vs*. 12.0 m, p = 0.002), whereas patients failed to obtain benefits from CCT in the “poor risk group” (14.0 *vs*. 12.0 m, p = 0.828). In view of the 1- and 3-year OS survival improved by CCT, but not 2- and 5-year OS rate of our conclusion, the results were not robust enough and the value of CCT is still worthy of further exploration.

The emergence of immunotherapy has changed the traditional treatment modalities of advanced cancers including EC. A multicenter, phase II, proof-of-concept study is ongoing to assess the efficacy and safety of atezolizumab in locally advanced ESCC patients who previously were treated with CCRT (25). The KEYNOTE-975 study (under recruitment) is a phase III randomized trial evaluating the efficacy of pembrolizumab in combination with definitive CCRT with fluorouracil and oxaliplatin or cisplatin in patients with locally advanced ESCC, EAC, and Siewert I adenocarcinoma of the gastroesophageal junction. Another ongoing phase III trial, BGB-A317-311, aims to assess the efficacy of tislelizumab *vs*. placebo in combination with CCRT and cisplatin and paclitaxel in localized ESCC. Relevant ongoing trials combining immunotherapy with CCRT in non-metastatic EC are represented in **Table 7**. These investigations provide new leads for the optimal treatment pattern for EC patients.

**Table 7:** Selected ongoing RCTs evaluating immunotherapy combined with CCRT and consolidation immunotherapy.

NCT number	Country	Phase	Histology	Start date	Estimated completion date	Study treatment	Estimated enrollment
NCT04426955	China	III	ESCC	2020	2022	CCRT ± concurrent and sequential SHR-1210	390
NCT03957590	China	III	ESCC	2019	2023	CCRT ± concurrent and sequential tislelizumab	316
NCT04210115	Global	III	ESCC, EAC	2020	2026	CCRT ± concurrent and sequential pembrolizumab	600
NCT04550260	US	III	ESCC	2020	2025	CCRT ± concurrent and sequential durvalumab	600

CCRT, concurrent chemoradiotherapy; EAC, esophageal adenocarcinoma; ESCC, esophageal squamous cell carcinoma; RCT, randomized controlled trial.

Our meta-analysis had some limitations. Firstly, as these studies were searched among published articles written in English, and statistical analysis was limited to the published data, language bias may exist. Secondly, nine of 10 included studies were retrospective studies, with the one prospective randomized study only including 110 cases. It was a pity that no randomized trial and prospective study with large cases has been published yet. Thirdly, we failed to perform subgroup analyses based on tumor stage, chemotherapy regimens, and radiation doses. As is well known, clinical stage is an independent prognostic factor. In our meta-analysis, approximately 10%–16% of the EC patients with stage I and stage IV were involved; although this factor of staging seems to be controlled and well balanced between the two groups in the original studies, this confounding factor still remains to be controlled in future studies. By far, there is still no consensus reached whether chemotherapy regimens influenced survival for EC patients with definitive concurrent chemoradiotherapy (dCRT) or not (26, 27); nevertheless, chemotherapy regimen and cycles of IC, CCT, and CCRT were inconsistent in our meta-analysis, which might have some potential impact on the result. Besides, we conducted sensitivity analysis in order to explore heterogeneity of dose delivery; after deleting each article in turn and reanalyzing the remaining research, the new results were consistent with the original results. In addition, the recently published randomized studies (28, 29) showed that no differences in OS were observed between 50 and 60 Gy.

In conclusion, the study revealed that additional IC or CCT with CCRT could prolong short-term survival in unresectable EC patients. Besides, the addition of CCT could significantly reduce the risk of distant metastases. As all patients were diagnosed with ESCC in the CCT-relevant studies, whether the conclusion of CCT was also applicable to patients with EAC requires further verification.

## Data Availability Statement

The datasets presented in this study can be found in online repositories. The names of the repository/repositories and accession number(s) can be found in the article/supplementary material.

## Author Contributions

All authors read and approved the final manuscript prior to submission. JuW and QP conceived and designed the study. SW and JiW contributed to data collection. JiW, SW, and LX performed the study and analyzed the data. JiW and LX wrote the paper. JuW and QP supervised the entire study and reviewed the final paper. All authors contributed to the article and approved the submitted version.

## Conflict of Interest

The authors declare that the research was conducted in the absence of any commercial or financial relationships that could be construed as a potential conflict of interest.

## Publisher’s Note

All claims expressed in this article are solely those of the authors and do not necessarily represent those of their affiliated organizations, or those of the publisher, the editors and the reviewers. Any product that may be evaluated in this article, or claim that may be made by its manufacturer, is not guaranteed or endorsed by the publisher.

## Abbreviations

CCRT, concurrent chemoradiotherapy; EC, esophageal cancer; ESCC, esophageal squamous cell cancer; EAC, esophageal adenocarcinoma; IC, induction chemotherapy; CCT, consolidation chemotherapy; OS, overall survival; LRR, local recurrence rate; DMR, distant metastasis rate.
